# Application of Visible/Near-Infrared Spectroscopy and Hyperspectral Imaging with Machine Learning for High-Throughput Plant Heavy Metal Stress Phenotyping: A Review

**DOI:** 10.34133/plantphenomics.0124

**Published:** 2023-11-30

**Authors:** Yuanning Zhai, Lei Zhou, Hengnian Qi, Pan Gao, Chu Zhang

**Affiliations:** ^1^ School of Information Engineering, Huzhou University, Huzhou 313000, China.; ^2^ College of Mechanical and Electronic Engineering, Nanjing Forestry University, Nanjing 210037, China.; ^3^ College of Information Science and Technology, Shihezi University, Shihezi 832003, China.

## Abstract

Heavy metal pollution is becoming a prominent stress on plants. Plants contaminated with heavy metals undergo changes in external morphology and internal structure, and heavy metals can accumulate through the food chain, threatening human health. Detecting heavy metal stress on plants quickly, accurately, and nondestructively helps to achieve precise management of plant growth status and accelerate the breeding of heavy metal-resistant plant varieties. Traditional chemical reagent-based detection methods are laborious, destructive, time-consuming, and costly. The internal and external structures of plants can be altered by heavy metal contamination, which can lead to changes in plants’ absorption and reflection of light. Visible/near-infrared (V/NIR) spectroscopy can obtain plant spectral information, and hyperspectral imaging (HSI) can obtain spectral and spatial information in simple, speedy, and nondestructive ways. These 2 technologies have been the most widely used high-throughput phenotyping technologies of plants. This review summarizes the application of V/NIR spectroscopy and HSI in plant heavy metal stress phenotype analysis as well as introduces the method of combining spectroscopy with machine learning approaches for high-throughput phenotyping of plant heavy metal stress, including unstressed and stressed identification, stress types identification, stress degrees identification, and heavy metal content estimation. The vegetation indexes, full-range spectra, and feature bands identified by different plant heavy metal stress phenotyping methods are reviewed. The advantages, limitations, challenges, and prospects of V/NIR spectroscopy and HSI for plant heavy metal stress phenotyping are discussed. Further studies are needed to promote the research and application of V/NIR spectroscopy and HSI for plant heavy metal stress phenotyping.

## Introduction

Due to industrial development and human activities, heavy metal pollution has become one of the primary pollutants in air, water, and soil. Previous research has shown that heavy metals ingested from plants may be transferred to the food chain and have adverse effects on human health based on their carcinogenicity [1]. According to reports, the risk of all cancer deaths increases due to long-term environmental exposure to cadmium and lead [2]. Heavy metals are one of the plants’ principal abiotic stresses, which hinder plant growth. Food shortages caused by climate/environment and political problems have been the main threat to the global food supply. With the continuous increase in population and the relative stability of cultivated land, the pressure on crop yield has prompted researchers to develop varieties with higher yield and stress resistance characteristics and has prompted farmers and growers to adopt advanced technologies to accurately monitor and manage plant growth.

Heavy metals have an impact on plant roots and aboveground parts. Research shows that heavy metal stress affects roots’ morphology and physiological and biochemical indicators [3,4]. The root growth morphology of plants is mainly affected by the inhibition of root length and root hair quantity, which hinders plants’ absorption of water and nutrients. The changes in physiological and biochemical indicators manifest as the activities of some antioxidant enzymes (including catalase and glutathione peroxidase) are inhibited or promoted, thus affecting the metabolic process and growth and development of plants [5]. Heavy metals also impact the aboveground parts of plants, typically manifested in their morphology, such as plant height and color. Usually, plant dwarfing, leaf deformation, and changes in leaf color occur [6,7]. In plants stressed by heavy metals, the physiological and biochemical indicators of the aboveground parts are affected by a decrease in pigment content, inhibition of photosynthesis, and partial reductase activity [8,9].

The external and internal structures of plants undergo changes under the stress of heavy metals, as well as changes in optical properties, which are reflected in the spectral characteristics of plants. Atomic absorption spectrometry, atomic fluorescence spectrometry, and inductively coupled plasma emission spectrometry are commonly used for the direct detection of heavy metal types and contents [10]. However, these methods are laborious, reagent-based, require complex sample preparation and instrument operation requirements, and have low efficiency. Indirect methods commonly used to assess heavy metal stress in plants include observing plant growth by experienced experts or farmers or measuring physiological and biochemical changes in plants using traditional methods. These methods are unsuitable for rapid, accurate, large-scale screening of plant heavy metal stresses.

In order to optimize plant growth management and improve breeding cycle and efficiency, high-throughput phenotype analysis technology has been widely used to rapidly, nondestructively, and effectively obtain plant phenotypes and to detect and analyze plants under heavy metal stress in recent years. Plant phenotype results from the interaction between genotype and environment, covering all morphological, physiological, and biochemical characteristics of plants. These features can be used to determine plant structure, composition, and growth. High-throughput measurement of plant phenotype helps to provide plant growth information, which can help to precisely monitor and manage plant growth and accelerate the breeding process. Based on the obtained plant phenotype, the growth status of plants can be accurately checked, providing guidance for plant growth management. Visible/near-infrared (V/NIR) spectroscopy and hyperspectral imaging (HSI) are the most widely used techniques in high-throughput plant phenotype analysis. V/NIR spectroscopy (350 to 2,500 nm) can obtain spectral information related to plants’ color, physiological and biochemical characteristics. HSI not only acquires spectral information as V/NIR spectroscopy but also obtains color, morphological, textural, and structural features.

**Fig. 1.**
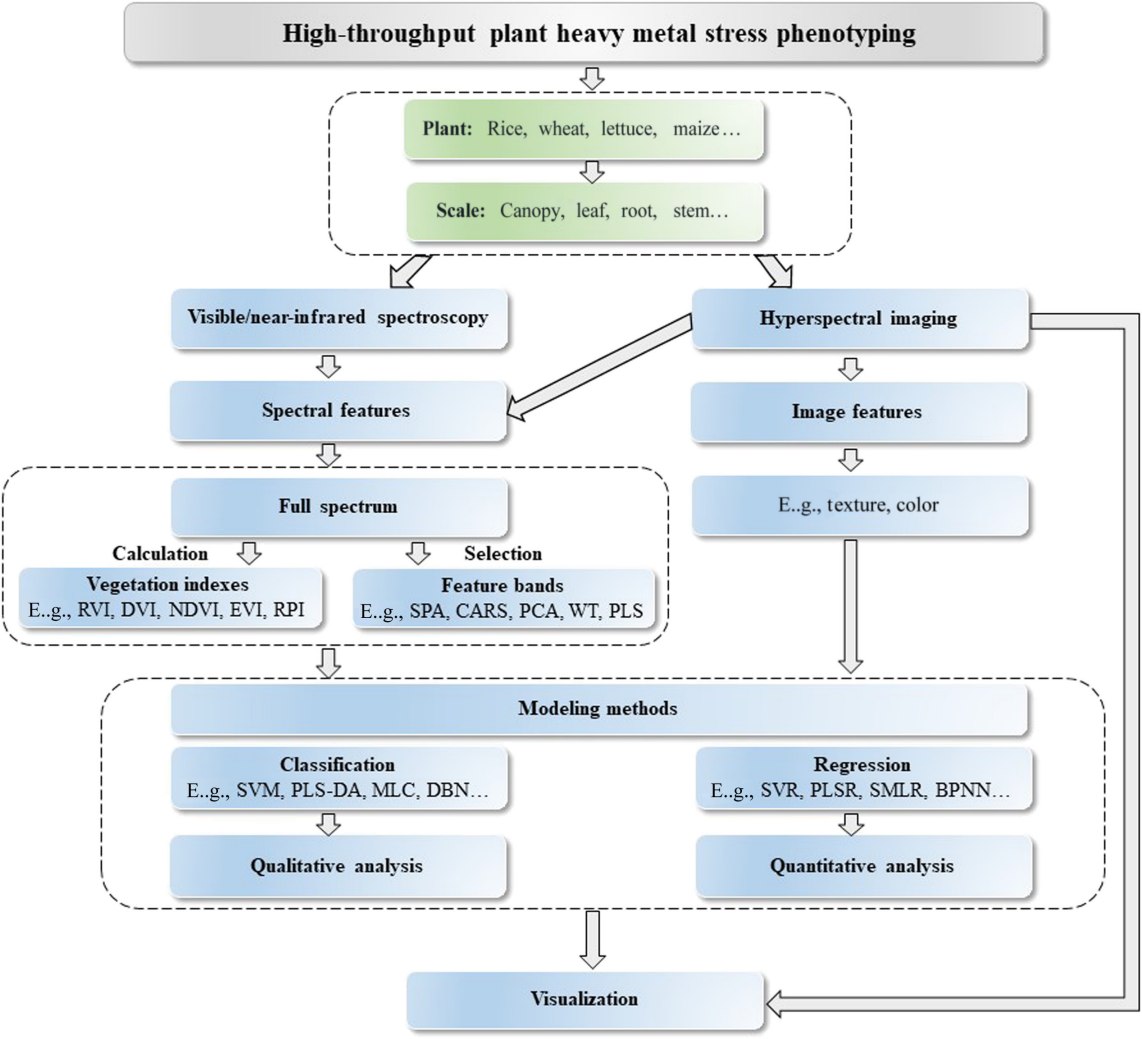
Summary of phenotypic analysis of plant heavy metal stress.

Since V/NIR spectroscopy and HSI are used for phenotyping information acquisition, various types of phenotype data can be used for analysis, such as vegetation index, full-range spectra, selected feature bands, etc. Machine learning methods are used to fully analyze the information of the phenotype data. This review aims to conduct a comprehensive summary of the applications of V/NIR spectroscopy and HSI for high-throughput phenotyping of plants under the stress of heavy metal with the help of machine learning methods. The widely used types of phenotype data, as well as the machine learning methods based on these types of data, were summarized and introduced. The steps for qualitative and quantitative analysis of plants contaminated with heavy metals as assessed using high-throughput phenotyping techniques are shown in Fig. 1. Furthermore, the advantages, limitations, challenges, and prospects of V/NIR spectroscopy and HSI for high-throughput phenotyping of plants under the stress of heavy metal were also discussed.

## Technical Principles and Spectral Profiles in Plant Heavy Metal Stress Inspection

### Technical principles of V/NIR spectroscopy and HSI

**Fig. 2.**
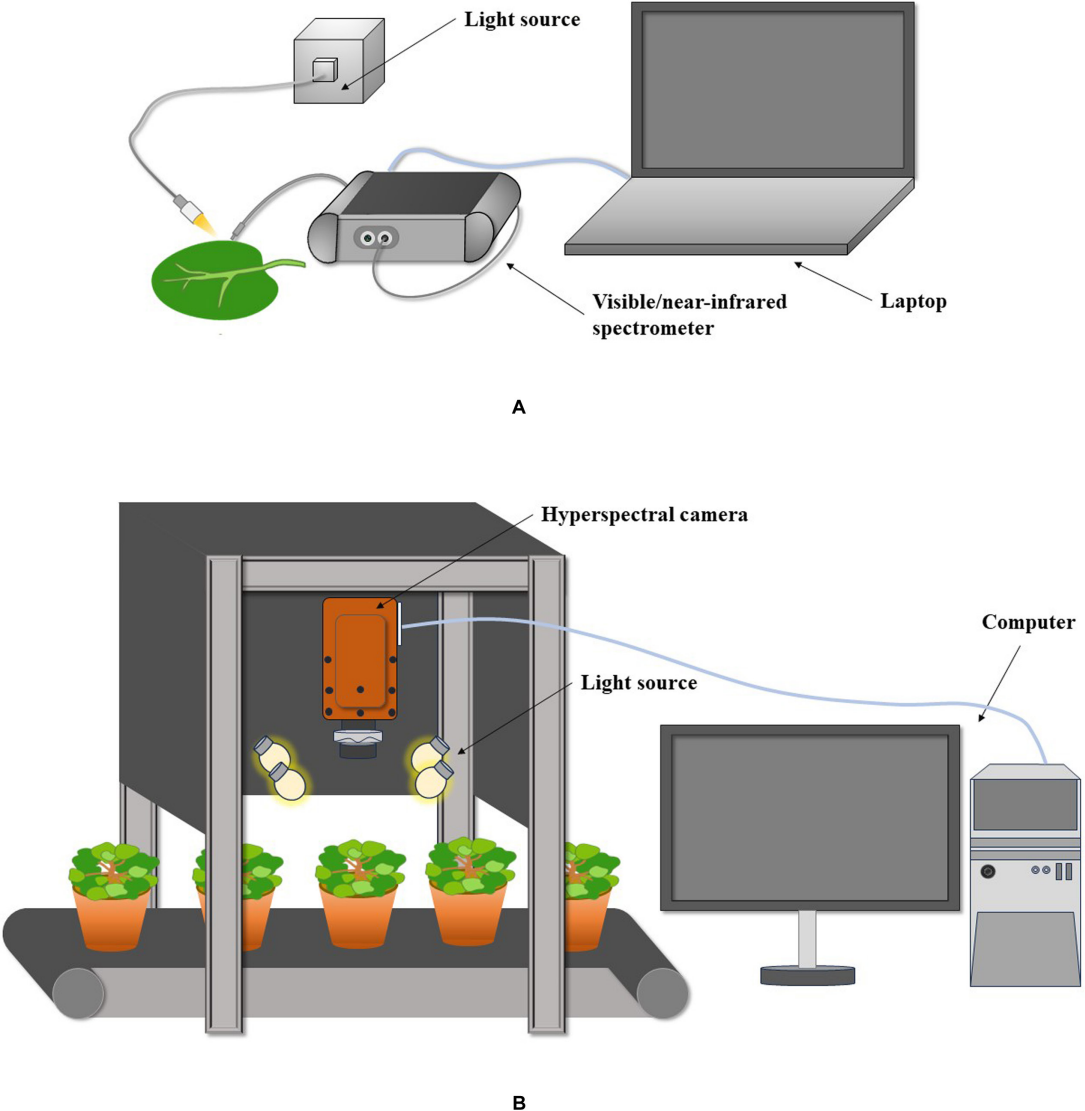
The schematic experimental setups of (A) V/NIR spectroscopy and (B) HSI system.

The principle of V/NIR spectroscopy technology is that when a sample is irradiated by light, a portion of the incident light is transmitted, absorbed, or reflected, causing bond vibrations between atoms. The V/NIR spectroscopy technique is based on the radiative absorptivity of the sample’s O-H, N-H, and C-H groups at molecular vibrational frequencies [11]. Absorbance refers to the amount of light absorbed by a sample, and changes in the water content and nutrient content of a sample can affect its absorbance, leading to changes in the absorption spectrum and intensity of the sample [12]. The range of V/NIR spectroscopy is 350 to 2,500 nm, with 350 to 780 nm in the visible light range and 780 to 2,500 nm in the near-infrared range. This technology does not require sample processing and can quickly and nondestructively detect the chemical and biological components of the sample. The schematic experimental setup of V/NIR spectroscopy is shown in Fig. 2A.

HSI captures visible and near-infrared spectra by measuring the light reflected or transmitted from the sample. A hyperspectral image is a cube that includes 1-dimensional spectral information and 2-dimensional spatial data, from which features such as texture, color, and shape of the sample can be obtained. HSI combines the advantages of spectroscopy and imaging techniques to provide high-resolution spectral and spatial information on samples and to obtain their physical and chemical features [13]. This technology can perform nondestructive analysis and evaluation of sample quality and is widely used in agriculture, food industry, and other fields. The schematic experimental setup of HSI system is shown in Fig. 2B.

### Spectral profiles of the plant under heavy metal stress

The spectral properties of plants are mainly determined by pigments, cell structure, and water content, and spectral reflectance can reflect the biochemical changes and growth status of plants. Plants absorb heavy metal elements from the soil through the root system, and the structure and chemical composition of the plant change, resulting in a change in its reflectance spectrum. Since spectra can reflect the vibrational information of molecular groups in plant tissue, many studies that detect heavy metal stress rely on changes in spectral reflectance. The color changes of plants are reflected in the visible light region (400 to 700 nm), and heavy metal contamination can be detected on the basis of spectral changes. Different pigments have different absorption spectral ranges, with carotenoids producing absorbance changes in the 420- to 503-nm range and anthocyanins in the 530- to 550-nm range. The range of leaf structure affecting plant spectral characteristics is 750 to 1,300 nm, and the range of water content affecting plant spectral characteristics is 1,300 to 2,400 nm. The visible light region is dominated by the spectral response of photosynthetic pigments, while the near-infrared region reflects structural properties, with water content dominating in the shortwave infrared region.

**Fig. 3.**
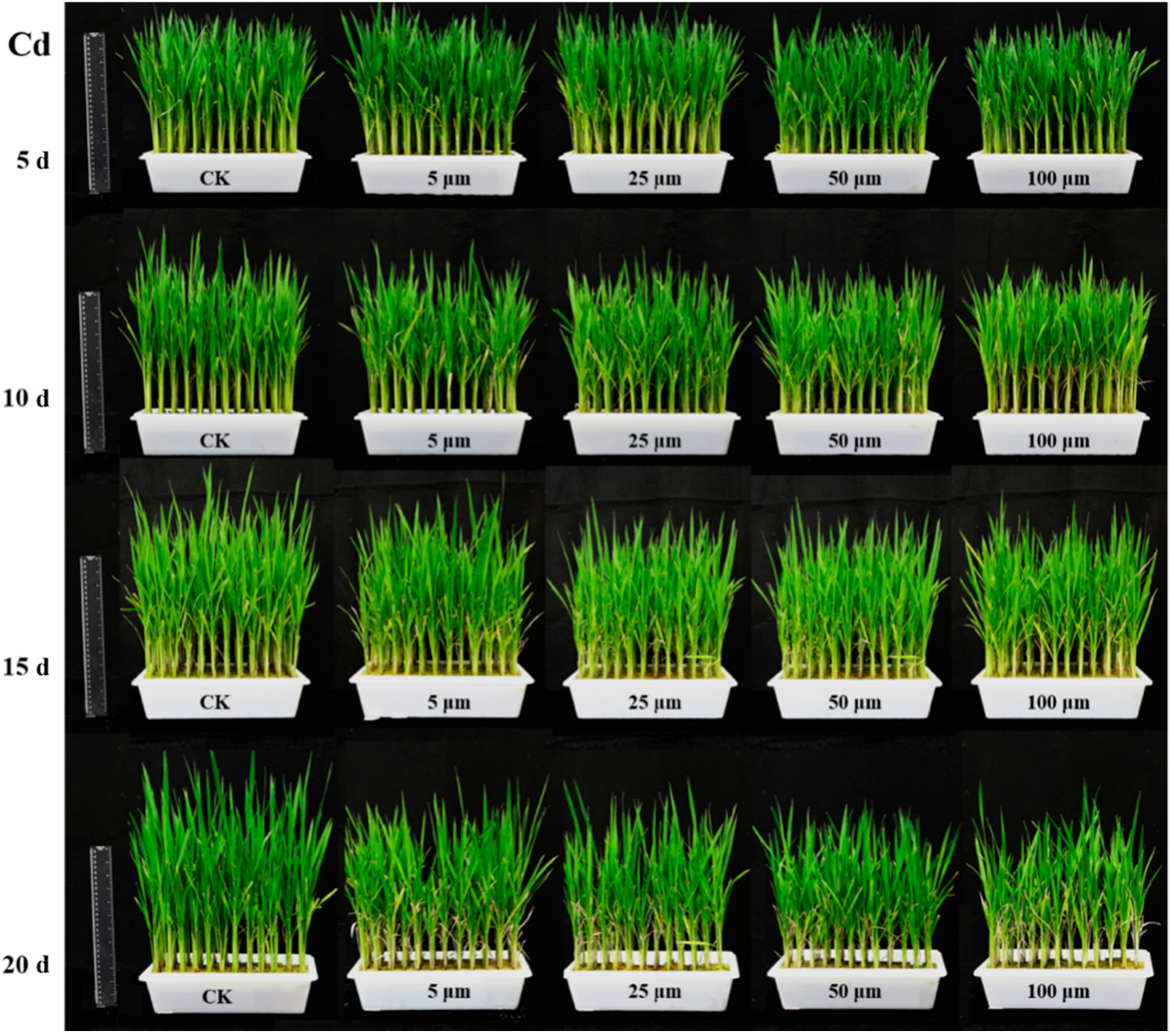
The growth status of rice plants under different contents of cadmium stress. Source: [16].

**Fig. 4.**
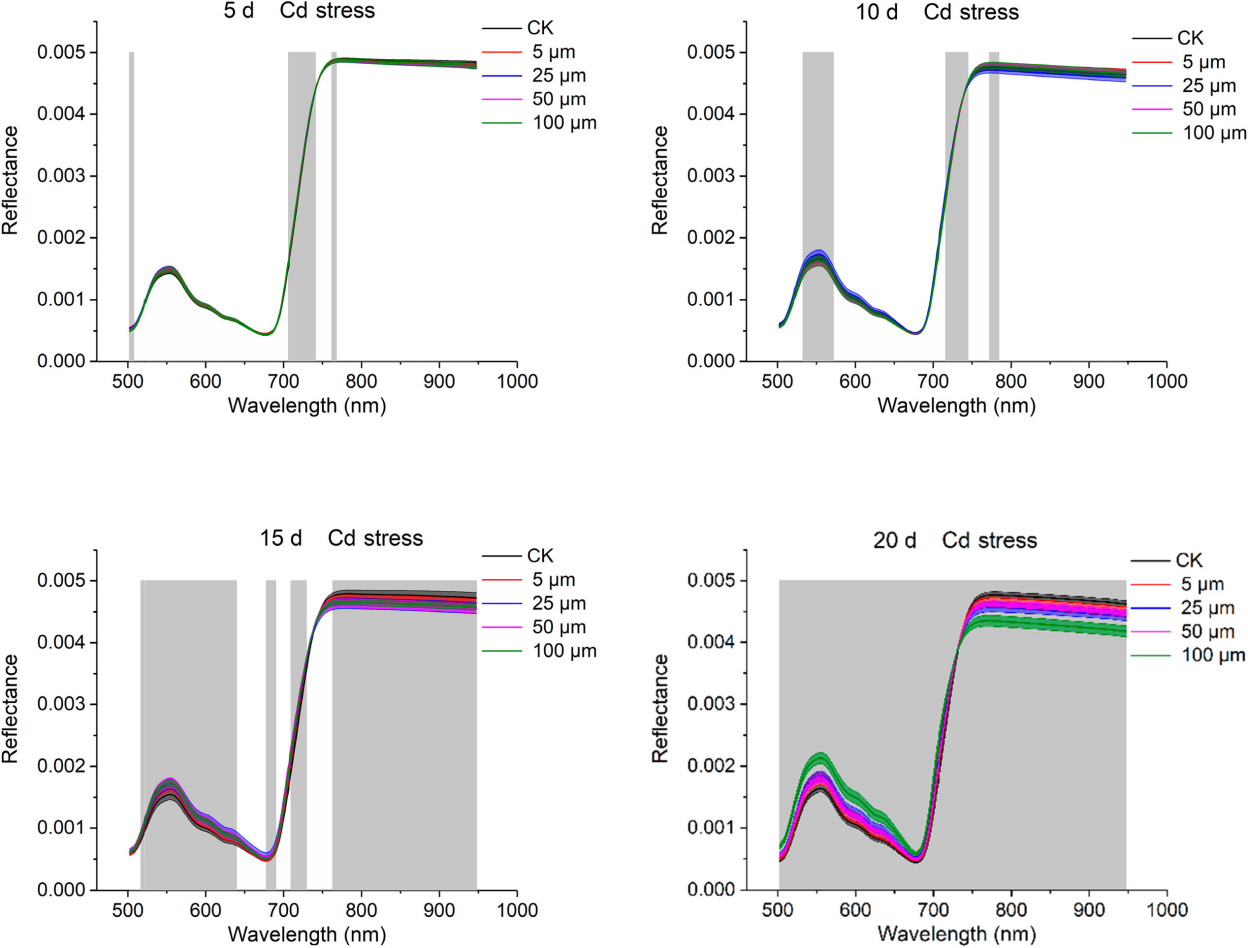
The changing trend of spectral curves of rice leaves under the stress of different contents of cadmium with increasing stress time. The gray area indicates obvious differences in the corresponding wavelengths. Source: [16].

The red edge position (REP) in a plant’s reflectance spectrum has the largest slope and is often used to estimate chlorophyll content. When vegetation is lush and chlorophyll is abundant, REP will move to a longer wavelength, while when vegetation is under stress, REP will move to a shorter wavelength. Plants were subjected to different levels of heavy metal stress, and the spectra showed similarity in the red edge region, with the REP values of plants subjected to heavy metal stress being prominently lower than those of control plants [14]. Similar to previous research results, the research results of Jun et al. [15] indicate that the heavy metal cadmium had a strong effect on chlorophyll content, and the wavelength band around 550 nm was effective in reflecting the chlorophyll content in leaves. Feng et al. [14] also studied the spectral response of plants under cadmium stress; for *Miscanthus sacchariflorus* leaf and root samples treated with different cadmium contents, the wavelength differences are even more pronounced in the visible light region. At the same time, Shen et al. [16] also studied the spectral differences of rice leaves at different times under cadmium stress. Figure 3 shows the growth status of rice plants under the stress of different contents of cadmium for 5, 10, 15, and 20 d. As the stress time increased, more spectral bands showed obvious differences. The spectral curve of rice leaves under cadmium stress is shown in Fig. 4. The trend of spectral changes with increasing stress time can be observed. Different from most studies on cadmium stress and chlorophyll changes in plants, Lassalle et al. [17] conducted on *Rubus fruticosus* L. under zinc stress. The results show that the increase of reflectivity at about 650 nm is usually attributed to the redness of leaves, which is due to the synthesis of anthocyanidin.

The light absorption and reflection performance of plants is affected by cellular structural damage, which alters the reflection and refraction pathways of light in plants, resulting in different spectral reflectance. The decrease in infrared reflectance caused by heavy metal stress is related to changes in leaf structure. Some studies analyze spectral changes caused by cell structure and water content. In addition, mature Norwegian spruce forests with similar ages were analyzed for stress from heavy metals. When studying the trend of spectral changes in plant canopy under stress, differences in water absorption characteristics centered around 985 and 1,178 nm were observed [18]. Moreover, the spectral differences are also manifested in wavelengths related to water (1,916 nm), starch (2,000 and 2,274 nm), and lipids (2,310 and 2,348 nm) [19]. The spectral variation bands of different plants under different heavy metal stresses are shown in Table 1.

**Table 1.** Summary of spectral changes in plant heavy metal stress

**Plant species**	**Heavy metal stress**	**Changing wavebands**	**Instrument spectral range**	**Study area**	**Reference**
Mustard spinach	Hg	550, 680	400–2,300 nm	Leaf	[58]
*Miscanthus sacchariflorus*	Cd	550, 680–750	380–1,030 nm	Leaf and root	[14]
Tomato	Cd	500–650, 710–760	431.05–962.45 nm	Leaf	[15]
Rice	Zn	460–680, 680–740, 970	350–1,050 nm	Leaf	[34]
Chicory	Cu	735, 910, 1,010, 1,105, 1,194, 1,380, 1,590	350–2,500 nm	Leaf	[65]
Rice	Cu, Cd	500, 670, 700, 800	350–2,500 nm	Leaf	[54]
*Brassica campestris* L.	Cu	460–670, 750–1,000	350–2,400 nm	Leaf	[66]
Sugar maple	Cu, Mn	475, 550, 660, 700, 775, 1,000, 1,475, 1,650	425–2,100 nm	Leaf	[67]
Rice	As	500, 680, 730, 1,450, 1,900	350–2,500 nm	Leaf	[68]
Lettuce	Pb	480, 550, 670	400–1,000 nm	Leaf	[61]
Wheat	Cu	543, 738, 741, 743	350–1,000 nm	Canopy	[31]
Lettuce	Cd	450, 550, 670, 760, 910, 960	380–1,030 nm	Leaf	[39]
Tobacco	Hg	550, 680–740, 970, 700–1,000	380–1,030 nm	Canopy and leaf	[7]
Rice	Cd, Pb	Cd: 681–776, 1,224–1,349; Pb: 712–784	350–2,500 nm	Leaf	[69]
Brown rice	Cd	540–570, 680–750	300–1,150 nm	Leaf	[70]
Lettuce	Cd, Pb	450, 550, 670–760, 750–900, 900–980	400.68–1,001.61 nm	Leaf	[53]
Lettuce	Pb	685–690, 730–740	480.46–1,001.61 nm	Leaf	[71]
Lettuce	Cd	685–690,730–740	480–1,010 nm	Leaf	[45]

## Spectral Features for Plant Heavy Metal Stress Inspection

### Vegetation indexes for plant heavy metal stress inspection

The ratio vegetation index was the earliest proposed vegetation index and is widely used to estimate and monitor green biomass and is closely related to plant biomass. The subsequently proposed differential vegetation index (DVI) is sensitive to soil changes and can be used to monitor the state of the growing environment of vegetation. The most widely used vegetation index currently is the normalized differential vegetation index (NDVI). NDVI values can reflect changes in plant chlorophyll content and vegetation coverage and represent plant growth status or vitality. Although NDVI is the most popular index for vegetation assessment, it does not necessarily mean that it is universally effective [20]. NDVI cannot distinguish plants treated with different metals [21]. If NDVI cannot meet the needs of vegetation assessment or other purposes, other vegetation indices can be considered. In previous studies, many vegetation indices have been developed for diagnosing plant growth. Zeng et al. [22] summarized 60 widely used vegetation indices and spectral ranges. Radocaj et al. [23] introduced 8 main vegetation indices based on multispectral sensors. Wang et al. [24] summarized the main vegetation indices for studying heavy metals.

The vegetation index is a useful and simple method that can be used to qualitatively and quantitatively evaluate vegetation vitality and growth dynamics. Assessing crop growth and health status through vegetation indices can provide information for detecting heavy metal stress in various plants and precision agriculture practices. Different vegetation indices can evaluate plant growth and development indicators, such as chlorophyll content, leaf area, and water status [23]. A study has shown a correlation between vegetation index and heavy metal contents, such as Ni, Cd, Zn, and Pb, with a Pearson correlation coefficient greater than 0.8 [25]. Researchers usually combine spectral data in different ways depending on the specific research objectives. Each vegetation index combines at least 2 bands, and the same index may involve a combination of various bands, depending on the detection objectives of the study and the level of heavy metal contents [26].

Many studies have utilized vegetation indices to assess the influence of heavy metal pollution on plant growth conditions. Zhang et al. [27] not only analyzed and compared traditional vegetation indices but also proposed a new vegetation heavy metal pollution index in order to qualitatively analyze the degree of pollution of different maize varieties under heavy metal copper stress. The new vegetation index has the advantages of simple calculation and high validity. Due to the error in using a single parameter indicator to evaluate the degree of heavy metal pollution, Wang et al. [28] attempted to simultaneously consider changes in chlorophyll, water, and nitrogen content in rice, in order to classify the extent of heavy metal stress in plants. In the study of the plant canopy, to evaluate rice in areas with different levels of heavy metal pollution, the vegetation index used was green normalized difference vegetation index, and a new normalized heavy metal stress index was proposed [29].

In addition, the use of vegetation indices to detect heavy metal contents in plants has been widely applied. Zhou et al. [30] studied wheat under Cu and Ni stress. The study analyzed the relationship between 19 common vegetation indices and heavy metal content in plants. The experimental results indicate that NPCI and normalized difference water index have the best correlation coefficients for nickel and copper. Unlike most studies on leaves, Wang et al. [31] discussed the relationship between increased copper content and canopy spectral reflectance. The study compared various vegetation indices, such as NDVI, modified triangle vegetation index, and NDVI/structure insensitive pigment index. The experimental results showed that NDVI/structure insensitive pigment index and W728 had the best prediction effect on the copper content in the wheat canopy during the tillering stage.

According to different research needs, many new vegetation indices have been proposed, but many have not been widely applied. Various vegetation indices reflect different vegetation characteristics and growth states. When analyzing plants under heavy metal stress, it is necessary to select appropriate vegetation indices based on the characteristics of the site and research purposes to obtain more accurate results. The common vegetation indices in existing research are shown in Table 2.

**Table 2.** Summary of research on vegetation index

**Vegetation index**	**Formula**	**Application**	**Reference**
NDVI	(*R*_800_ − *R*_670_)/(*R*_800_ + *R*_670_)	Chlorophyll	[72]
RENDVI	(*R*_750_ − *R*_705_)/(*R*_750_ + *R*_705_)	Chlorophyll	[73]
MRENDVI	(*R*_750_ − *R*_705_)/(*R*_750_ + *R*_705_ − 2 × *R*_445_)	Chlorophyll	[74]
EVI	2.5 × ((*R*_800_ − *R*_670_)/(*R*_800_ − (6 × *R*_670_) − (7.5 × *R*_475_) + 1))	Chlorophyll	[75]
OSAVI	(1 + 0.16) × (*R*_800_ − *R*_670_)/(*R*_800_ + *R*_670_ + 0.16)	Chlorophyll	[76]
MTVI	1.5 × [1.2 × (*R*_712_ − *R*_550_) − 2.1 × (*R*_670_ − *R*_550_)]	Chlorophyll	[54]
MCARI	[(*R*_700_ − *R*_670_) − 0.2 × (*R*_700_ − *R*_550_)] × (*R*_700_/*R*_670_)	Chlorophyll	[54]
NRI	(*R*_570_ − *R*_670_)/(*R*_570_ + *R*_670_)	Nitrogen	[77]
PSRI	(*R*_690_ − *R*_500_)/*R*_550_	Senescence	[78]
ARI	(*R*_550_ − 1)/(*R*_700_ − 1)	Anthocyanin	[79]
SIPI	(*R*_800_ − *R*_445_)/(*R*_800_ − *R*_680_)	Cell structure	[80]
PRI	(*R*_531_ − *R*_570_)/(*R*_531_ + *R*_570_)	Cell structure	[81]
WI	(*R*_870_/*R*_950_)	Water content	[82]
WBI	(*R*_900_/*R*_970_)	Water content	[83]
DSWI	(*R*_803_ + *R*_549_)/(*R*_1, 659_ + *R*_681_)	Water content	[84]
MSI	(*R*_1, 599_/*R*_819_)	Water content	[85]
NDWI	(*R*_860_ − *R*_1, 240_)/(*R*_860_ + *R*_1, 240_)	Water content	[86]

MRENDVI, modified red edge normalized difference vegetation index; EVI, enhanced vegetation index; OSAVI, optimized soil-adjusted vegetation index; MTVI, modified triangle vegetation index; MCARI, modified chlorophyll absorption ratio index; NRI, nitrogen reflectance index; PSRI, plant senescence reflectance index; ARI, anthocyanin reflectance index; SIPI, structure insensitive pigment index; PRI, photochemical reflectance index; WI, water index; WBI, water band index; DSWI, disease stress water index; MSI, moisture stress index; NDWI, normalized difference water index

### Full spectrum for plant heavy metal stress inspection

When analyzing plants under heavy metal stress, full-spectrum modeling is a common method used to fully utilize the abundant information contained in spectral data. By obtaining data from plant samples across the entire spectrum range, models can be built for qualitative analysis. Qualitative analyses usually involve determining the degree and type of stress in a plant sample and classifying it as stressful or nonstressful. The qualitative analysis aims to provide decision-makers and researchers with a fast, accurate, and reliable method to determine whether plant samples are subjected to heavy metal stress. In quantitative analysis, the objective is to determine the content of heavy metals or physiological and biochemical components in plant samples. Full-spectrum modeling provides a nondestructive and high-throughput method for quantitative analysis. By modeling the spectral features related to known heavy metal content, a quantitative relationship between the spectrum and heavy metal content can be established. The establishment of a full-spectrum model requires an adequate number of samples and provides an efficient and nondestructive analytical tool. It not only helps researchers understand the extent of heavy metal stress in plants but also provides valuable references for environmental protection, agricultural management, and soil remediation.

In the study of rice leaves under heavy metal stress, the experimental results showed that heavy metals had the most severe effect on early rice growth [32]. Some studies have shown that heavy metal elements are absorbed in various parts of rice, such as roots, stems, and leaves, with the roots being the most absorbent. In addition, a study investigated the content of inorganic arsenic in rice by collecting freeze-dried and ground samples, using full-spectrum data to establish a regression model for predicting inorganic arsenic content [33].

The regression model established based on the full-spectrum data of rice leaves can effectively predict the heavy metal content in rice leaves [34]. A study focused on the analysis of the heavy metal cadmium in rice, which can cause harm to rice growth and affect its physiological and metabolic processes [35]. Research has found that the zinc content in rice roots and leaves increases with the increase of zinc content in the soil, and the length of rice roots is inversely proportional to zinc content. The research on full-spectrum modeling, the types of stress plants are subjected to and the research scale are listed in Table 3.

**Table 3.** Summary of research on full-spectrum modeling

**Plant species**	**Heavy metal stress**	**Study area**	**Reference**
*Seriphidium terrae-albae*	Cu	Canopy	[87]
Rice	Cd, Hg, Pb, As	Root	[88]
Norwegian spruce needle	Cu, Zn, As, Hg	Leaf and canopy	[18]
*Rubus fruticosus* L.	Cr, Cu, Ni, Zn	Leaf	[17]
Rice	Pb	Leaf and canopy	[32]
*Brassica campestris* L.	Cu	Leaf and root	[66]
Barley	Cd, Pb, As	Leaf	[89]
Salicornia	Cd, V	Leaf	[90]
Tobacco	Hg	Canopy	[7]
Brown rice	Cd	Leaf	[70]
*Ludwigia prostrata* Roxb	Cu, Zn	Leaf and root	[6]
Green tea	Pb	Leaf	[36]
Rice	Cd	Leaf	[16]
Rice	As	Leaf	[68]
Lettuce	Pb	Leaf	[61]
*Miscanthus floridulus* (Labill.) Warb	Hg	Stem and leaf	[56]

### Feature bands for plant heavy metal stress inspection

When analyzing plants under heavy metal stress, selecting appropriate spectral feature bands is an important step. By choosing feature bands that are highly correlated with the sample’s condition, the volume of spectral data can be reduced, improving the efficiency of processing and analyzing the data. Simultaneously, selecting feature bands helps eliminate interference factors in the spectral data, thereby improving the accuracy of subsequent models built based on the selected feature bands. A variety of methods are available for feature band selection, including machine learning methods (such as random forests, support vector machines [SVMs], etc.), feature extraction methods (such as wavelet transform, discrete cosine transform, etc.), and statistical methods (such as correlation coefficient method, principal component analysis [PCA], etc.). As shown in Table 4, successive projections algorithm (SPA), wavelet transform (WT), competitive adaptive reweighted sampling (CARS), partial least squares (PLS), and PCA are commonly used methods for feature band selection.

**Table 4.** Summary of research on selecting feature bands

**Plant species**	**Heavy metal stress**	**Select feature bands**	**Reference**
*Miscanthus sacchariflorus*	Cd	SPA, CARS	[14]
Maize	Cu, Pb	RDA	[91]
*Jatropha curcas*	Cu	PCA	[41]
Tomato	Cd	WT-LSSVR	[15]
Solanaceae plant	Ca, Mg	ICO	[92]
Chicory	Cu	PLS-VIP scores	[65]
Grape seedlings	Cu, Zn, Pb, Cr, Cd	PLS	[40]
Barley	Cd, Pb, As	CR	[89]
Lettuce	Cd	SPA	[37]
Wheat	Cu	Second derivative	[31]
Maize	Cu, Pb	Pearson	[93]
Rice	Cd, Pb	ANOVA	[69]
Lettuce	Cd	IRIV	[47]
Lettuce	Cd	WPCA	[45]
Lettuce	Cd	SPA, PLSR, SAE	[39]
Tobacco	Hg	PCA, CARS	[7]
Rice	Cd, Pb	ANOVA, Random forest	[94]
Rice	Cd, Pb	ANOVA	[50]
Lettuce	Cd, Pb	SPA, VISSA	[53]
Oilseed	Pb	MRF, CARS	[48]
Tobacco	Zn, As, Cd, Hg, Pb, Cr	UVE, CARS, Random frog	[95]
Rice	As	GA	[96]
Tomato	Cd	MC-siPLS	[44]

WT-LSSVR, WT and partial least squares support vector regression; RDA, ratio difference of autocorrelation function first derivative; ICO, interval combination optimization; PLS-VIP, partial least square-the variable important in projection; CR: continuum removal; ANOVA, two-way analysis of variance; IRIV, iteratively retaining informative variables; WPCA, wavelet principal component analysis; SAE, stacked autoencoder; VISSA, variable iterative space shrinkage approach; MRF, modified random frog; UVE, uninformative variable elimination; GA, genetic algorithm; MC-siPLS, synergy interval PLS couple with Monte Carlo method

In feature band selection, SPA and CARS methods are often employed for band screening, followed by model establishment using the extracted spectral data [14,36,37]. Wang et al. [38] used the wavelet decomposition method to extract detail coefficients and select feature bands for the maize leaf spectrum, achieving rapid maize screening under varying degrees of copper stress. Furthermore, in order to establish a more efficient and accurate model for detecting cadmium in tomato leaves, a method combining WT and PLS support vector regression was proposed to select effective feature bands. The optimal predictive model had the *R*^2^ of 0.8937 and root mean square error (RMSE) of 0.2331 mg/kg [15]. In comparison to other feature band selection methods, PLS exhibits good robustness and predictive performance, enabling the determination of optimal bands using PLS methods [39,40].

Yu et al. [7] compared PCA and CARS methods and showed that the CARS algorithm chose a more comprehensive band to identify the tobacco canopy under mercury stress. The accuracy of the classification model for distinguishing between stressed and nonstressed groups established through CARS selection variables was 100%. PCA is a commonly used statistical method for data dimensionality reduction, but its effectiveness in nonlinear data relationships may be limited. Linear discriminant analysis of different degrees of copper stress on the stems and roots of *Jatropha curcas* L. was studied using an optimal band based on PCA analysis, with a classification accuracy of 83.93% [41]. Some studies have improved the PLS method and combined it with other methods for feature band selection, such as the synergistic interval PLS (siPLS) algorithm, which enhances modeling predictive accuracy [42,43]. Furthermore, a recent study combined siPLS with the Monte Carlo method and proposed MC-siPLS, which was superior to similar wavelength interval selection methods. The optimal model had an RMSE of 0.5378 and an *R*^2^ of 0.9870 [44].

### Comparisons

Due to the complexity of different spectral instruments and resolutions used, there is no unified mathematical expression to define all vegetation indices. Vegetation index is a nondestructive monitoring method, but it also has some drawbacks, such as a lack of sensitivity. In addition, because of the variability and complexity of environmental factors, such as soil moisture, which can affect the accuracy of vegetation indices, the use of vegetation indices in practical applications needs to be taken into account.

Full-spectrum modeling can fully utilize the rich information contained in spectral data. At the same time, the problem of multicollinearity may occur and the accuracy of the full-spectrum modeling will be reduced. When applying full-spectrum modeling, attention should be given to issues such as sample selection, data processing, and model validation in order to build models with higher accuracy.

Selecting feature bands can eliminate confounding in spectral data, thus improving the accuracy of subsequent models based on feature bands. In addition, it also has the advantage of being more convenient for spectral data analysis and visualization. Due to the high redundancy of hyperspectral data, the successful selection of feature bands sensitive to heavy metals is particularly important.

In practical applications, it is necessary to consider and analyze different methods in an integrated manner, which can be used in combination depending on limitations such as environmental factors. Feature band modeling can select wavelengths with information and eliminate wavelengths without information. At the same time, it may lead to useful information being ignored, so it is usually necessary to establish a full-spectrum model for comparison in order to select the optimal result.

## Image Features for Plant Heavy Metal Stress Inspection

In diagnosing plants contaminated with heavy metals, HSI has a number of different applications and characteristics compared to V/NIR spectroscopy. HSI usually covers a wider spectral range and is usually higher in spectral resolution. It can provide tens to hundreds of continuous spectral bands, allowing for a more detailed analysis of the spectral characteristics of plant reflection and absorption. Hyperspectral images can provide detailed spatial details and capture the differences in heavy metal stress on plants at different locations. HSI has high spatial resolution and can provide spatial distribution analysis of heavy metal stress in plants. By acquiring a large amount of hyperspectral image data and combining it with spatial analysis methods, it is possible to map the spatial distribution of plants subjected to different levels of heavy metal stress. This helps to identify areas contaminated by heavy metals and provides information on the degree of stress and spatial changes, thereby guiding environmental monitoring and resource management decisions.

**Fig. 5.**
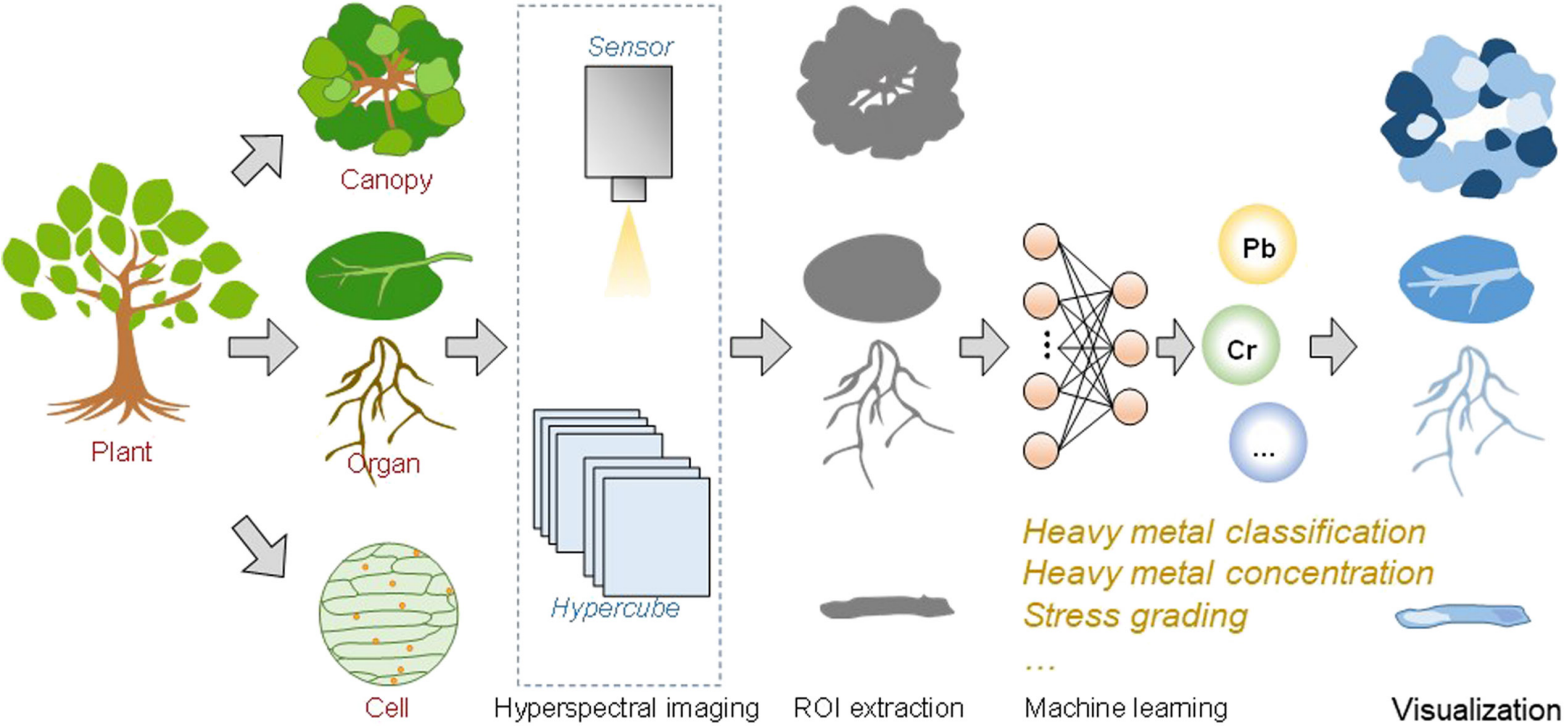
Steps for detecting heavy metal stress in plants based on HSI. ROI, region of interest.

Hyperspectral images allow the analysis of the heterogeneity of the chemical composition in the samples and enable real-time monitoring of heavy metal contamination over large areas. Zhou et al. [45] used fluorescence HSI to classify lettuce leaves subjected to different heavy metal cadmium stress levels. The classification accuracy based on the calibration and the prediction sets was 99.79% and 94.19%, respectively. In another study using fluorescence HSI on lettuce leaves, the heavy metals cadmium and lead were detected and the results were visualized, taking into account the effects of compound heavy metals. The results indicated that the *R*^2^ for predicting Cd content was 0.7905, and the *R*^2^ for predicting Pb content was 0.8965 [46]. Similarly, Zhou et al. [47] obtained hyperspectral images of lettuce leaves at different cadmium contents and visualized the predicted cadmium content in lettuce leaves. The *R*^2^ of the obtained optimal prediction model was 0.8843, and the RMSE was 0.1292 mg/kg. Cao et al. [48] used hyperspectral technology for nondestructive detection of Pb content in rapeseed leaves. The *R*^2^ and RMSE based on the prediction set were 0.9431 and 0.1645 mg/kg, respectively. The same research on rape combines HSI technology with ensemble learning methods to visually analyze the Cd content in oilseed rape leaves [49]. The *R*^2^ of the optimal model in this study was 0.9815, and the predicted RMSE was 5.8969 mg/kg. Feng et al. [14] separated the leaves and roots as target regions from the obtained hyperspectral images. Finally, the heavy metal cadmium content in different plant tissues was visualized on a prediction map. Yu et al. [7] obtained hyperspectral images of tobacco canopy and compared the appearance and microstructure of tobacco plants, achieving qualitative discrimination between heavy metal Hg stress and nonstress tobacco plants. In addition, the feasibility of using hyperspectral images to quickly diagnose cross-contamination of heavy metals Pb and Cd in rice was explored [50]. The study collected hyperspectral data from rice canopies, and the results showed that different degrees of heavy metal stress were successfully classified. The steps involved in HSI-based plant heavy metal stress detection methods are summarized in Fig. 5.

## Summary of Modeling Methods for Plant Heavy Metal Stress inspection

### Traditional machine learning methods

After multivariate analysis of the acquired spectral data, the choice of an appropriate modeling method is crucial. Traditional machine learning algorithms have been extensively studied and applied, often yielding good results. In the research of plant heavy metal stress, machine learning classification algorithms are commonly employed for qualitative analysis. These include SVM [28], PLS discriminant analysis (PLS-DA) [36], maximum likelihood classification [18], and extreme learning machine [51]. These algorithms learn spectral features and patterns from the training samples to predict the classification of new samples. These classification models were established to assess whether plants are under heavy metal stress, to assess the severity, and to distinguish between stress types. PLS regression (PLSR) [52], multiple linear regression (MLR) [40], support vector regression (SVR) [53], backpropagation neural network (BPNN) [54], and stepwise multiple linear regression (SMLR) [55] are widely used in quantitative analysis of plant heavy metal stress contents. These algorithms utilize the multivariate information of spectral data and training samples with known heavy metal content to build regression models for predicting the heavy metal content of unknown samples. Quantitative analysis can provide more accurate information on heavy metal contents, helping researchers and decision-makers better understand the extent of heavy metal stress in plants and take appropriate measures.

SVM is suitable for nonlinear classification. Wang et al. [28] investigated rice samples from 3 different pollution levels in farmland. The results demonstrated that the established SVM model successfully classified the rice samples according to pollution levels with high accuracy. PLS-DA is commonly used for classification and discrimination problems. By studying the changes in leaves under different levels of lead stress, a PLS-DA model was established to classify tea samples with an accuracy of 0.979 [36]. Similarly, Tang et al. [56] and Yu et al. [7] utilized the PLS-DA method to establish classification models for mercury pollution levels in Gramineae plants and tobacco, respectively. In the case of tobacco research, the PLS-DA model did not yield satisfactory results, prompting the research to adopt the least squares SVM method of classification to differentiate between contaminated tobacco plants. In addition to the above methods, Cui et al. [51] combined reflectance spectral data with the extreme learning machine method to construct a classification model for copper pollution, the accuracy of this model reached 89.02%.

Liu et al. [54] applied the BPNN model to monitor the chlorophyll content changes in rice leaves contaminated with heavy metals, and the optimal prediction model had an *R*^2^ of 0.9014 and an RMSE of 2.58. Furthermore, the study comprehensively evaluated the model performance by comparing it with statistical regression models. Similarly, Qing et al. [57] established predictive models for BPNN, PLSR, and SMLR using on-site canopy spectral data. By comparing the model results, it is found that BPNN is the best model selection. The SMLR method is usually used for high-dimensional datasets to determine the optimal combination of independent variables and enhance the predictive ability of the model. Dunagan et al. [58] used this approach to develop a regression model to quantify mercury in plant leaves. Similarly, Lamine et al. [55] and Zuzana et al. [59] also utilized the SMLR method to establish related models for predicting heavy metal content. Miao et al. [60] developed a PLSR model based on near-infrared spectroscopy combined with 4 chemometric methods to predict cadmium content in rice; the *R*^2^ and RMSE of biPLS model were 0.9020 and 0.2133, respectively. Some studies have proposed improvements to PLSR, such as the modified PLS regression algorithm and established corresponding models [19,33]. In the research on heavy metals in rapeseed, near-infrared hyperspectral technology combined with the SVR algorithm was employed to detect the lead content in rapeseed leaves, with further optimization of SVR parameters [48]. Similarly, Zhou et al. [47] utilized SVR to establish a model for predicting cadmium content in lettuce leaves.

### Deep learning methods

Deep learning, an emerging field in machine learning, has shown tremendous potential in addressing complex problems. Nowadays, deep learning methods have been widely employed to detect heavy metal stress in plants and have achieved promising results. This section discusses their application in heavy metal stress detection. In one study, discriminative models such as PLS-DA, SVM, and deep belief network (DBN) were developed respectively, and the final results showed that the best results were obtained by using DBN to detect lead stress levels in lettuce leaves. The accuracy of the DBN model was 100% in the training set and 96.67% in the test set [61]. Compared to traditional models, DBN models are more capable of learning and predicting, with more substantial feature extraction and shorter training time. Furthermore, the model can also be utilized for quantitative analysis of heavy metal stress. Sun et al. [37] conducted a similar study and employed 3 methods to establish regression analysis models: DBN based on particle swarm optimization (PSO), partial least squares regression, and SVR. In the study, the PSO algorithm was introduced in the pretraining stage to solve the connection weight. The PSO-DBN model was proven to be the best model for predicting cadmium content, with an *R*^2^ of 0.9234 and RMSE of 0.5423 mg/kg. Furthermore, a study proposed a transfer stacked autoencoder algorithm for detecting lead in oilseed rape by applying transfer learning to an optimal stacked autoencoder deep learning network; the best model for predicting lead content in oilseed rape leaves and roots had *R*^2^ values of 0.9215 and 0.9349, respectively [62]. Similarly, Zhou et al. [63] investigated lead contamination of oilseed rape leaves by combining HSI techniques with deep learning algorithms. This study was based on WT and stacked denoising autoencoder to extract the depth features, the *R*^2^ and RMSE of the established model were 0.9388 and 0.0199 mg/kg.

Currently, deep learning methods have demonstrated superior performance to traditional machine learning methods in practical applications, providing a new approach for detecting heavy metal stress in plants. Due to the large amount of data in hyperspectral images and the increased workload of data processing, the establishment of detection models becomes complex and costly. At this time, deep learning such as the DBN and convolutional neural network algorithms can be selected to extract the depth features of hyperspectral images. Moreover, the combination of deep learning and traditional machine learning methods can be employed to maximize model accuracy and achieve more effective detection of heavy metal stress in plants. The commonly used modeling methods are listed in Table 5.

**Table 5.** Summary of research on modeling methods

**Plant species**	**Heavy metal stress**	**Study area**	**Modeling methods**	**Reference**
Rice	Cu, Pb	Leaf	PLSR	[97]
Brown rice	Cd	Leaf	PLSR	[70]
*Miscanthus sacchariflorus*	Cd	Leaf and root	PLSR, LS-SVM	[14]
Barley	Cd, Pb, As	Leaf	PLSR, MLR	[89]
Mustard spinach	Hg	Leaf	SMLR	[58]
Grapevine foliage	Cu, Zn, Pb, Cr, Cd	Leaf	MLR, SVM	[40]
Rice	Cd, Pb	Canopy	SVM	[94]
Rice	Cd, Pb	Canopy	SVM	[50]
Lettuce	Cd	Leaf	SVM	[98]
Lettuce	Cd, Pb	Leaf	SVR	[53]
Lettuce	Pb	Leaf	SVR	[71]
Lettuce	Cd	Leaf	SVR	[47]
Lettuce	Pb	Leaf	SVR	[99]
Oilseed rape	Pb	Leaf	SVR	[48]
Tobacco	Hg	Canopy	PLS-DA, LS-SVM	[7]
Lettuce	Pb	Leaf	PLS-DA, SVM, DBN	[61]
Lettuce	Cd	Leaf	PLSR, SVR, PSO-DBN	[37]
Rice	Cu, Cd	Leaf	BPNN	[54]

## Challenges and Prospects

Currently, there are still some challenges in using high-throughput technology to analyze plant heavy metal stress phenotypes: (a) In most of the published articles for plant heavy metal stress analysis, the studies’ vegetation indexes were cited from previous literature. These well-known vegetation indexes were proved to be sensitive to water/nutrient content, which might be highly correlated with multiple plant stresses. Such vegetation indices were not specifically designed for heavy metal stress detection. (b) There are various kinds of algorithms for selecting important spectral wavelengths. Current studies focus on screening heavy metal-sensitive wavelengths which is suitable for a certain type of plant. There are few research cases, and it is very hard to systematically summarize. These factors all produce a negative impact on the diagnosis of heavy metal stress. (c) The existing studies have covered the experiments at different scales, including organ level and canopy level. “Which scale is more suitable for heavy metal stress detection?” requires further exploration. (d) Accurate detection of heavy metal stress requires extensive data support, including the collection of soil and plant samples, recording and analysis of experimental data, and more. This involves issues related to sampling accuracy and data quality. During the collection of V/NIR spectroscopy and hyperspectral image data, many factors, such as light intensity, imaging angle, instrument type, plant height, etc., will generate a certain response signal in the spectral image. Such responses might cause interference with heavy metal stress detection. (e) The current research is not systematic. The effects generated by different heavy metals, different plants, different cultivation methods, and different growth periods were rarely considered in the previous literature. For the heavy metal-stressed plants of different varieties and species, the differences and commonalities of their spectral responses were rarely explored. (f) Toxicity to plants caused by heavy metal stress is a complex process that is influenced by a variety of factors including soil, environment, and plant species. Accurately assessing and quantifying the contributions of different factors to heavy metal stress and their interactions remains a challenge.

With the rapid advancement of data acquisition technologies and the increasing availability of computational resources, the field of heavy metal stress analysis is expected to witness promising developments in the future. (a) New vegetation indexes, which are specifically for heavy metal stress-related plant phenotyping, should be studied. (b) Heavy metal stress-sensitive spectral wavelength screening algorithms need to be developed and improved. (c) Temporal phenotyping analysis methods for analyzing the dynamic response of plants under heavy metal contamination should be created and investigated, which could be a potential solution for overcoming abnormal symptomatic disturbances induced by other stresses or disturbances in phenotypic changes caused by natural growth processes. (d) The methods for removing the spectral response caused by the variations of sensors and measuring conditions should be considered. (e) Differences and commonalities of the spectral responses generated by the plants varying from varieties and species should be studied and summarized. (f) New data processing algorithms for heavy metal stress-related plant phenotyping, such as deep learning-based image processing, transfer learning, and meta-learning, are expected to be designed. Meanwhile, to support the establishment of deep learning model training, large dataset collection approaches and devices should also be designed.

The fusion of multiple data sources, including spectral data, remote sensing data, and environmental monitoring data, holds great promise in providing more comprehensive and accurate information about heavy metal stress [64]. Combining deep learning techniques with feature engineering and machine learning methods may play a crucial role in improving the performance and interpretability of heavy metal stress models. In summary, by combining multiple data sources and advanced technologies and continually improving models and algorithms, it is expected to achieve more accurate, efficient, and sustainable detection and management of heavy metal stress in plants. This will have positive impacts on agricultural production, environmental protection, and food safety, among other fields.

## Conclusions

This review summarizes the application of HSI and nonimaging V/NIR spectroscopy techniques in plant heavy metal stress phenotype analysis in recent years and analyzes and compares the effects of different plants under heavy metal stress. The common types of heavy metal pollution currently include cadmium, copper, lead, zinc, mercury, etc. The pollution of heavy metals on plants seriously affects their growth and development processes, posing a threat to human health. The vegetation index is an essential indicator of plant growth status and is used to analyze the level of heavy metal contamination of plants. The establishment of heavy metal stress detection models is usually divided into full-spectrum modeling and feature band modeling. Full-spectrum modeling covers the information of all bands, but there is a multicollinearity problem. Feature band modeling can avoid this problem, but sometimes it ignores potentially useful information. In order to obtain highly accurate models, multiple methods are usually used for comparison, and multiple approaches can also be combined to build models. Commonly used machine learning and deep learning methods can achieve good results. In summary, V/NIR spectroscopy and HSI technologies have a broad range of application scenarios in the analysis of plant heavy metal stress phenotypes. In the future, multiple sensing technologies and modeling methods can be combined to improve the stability and applicability of the detection model, further promote the integration of technology and practical applications, achieve early detection and accurate diagnosis of plant heavy metal stress, and contribute to ensuring human health and ecological environment safety.
